# Upregulation of POC1A in lung adenocarcinoma promotes tumour progression and predicts poor prognosis

**DOI:** 10.1111/jcmm.18135

**Published:** 2024-03-01

**Authors:** Zi‐Hao Li, Jia‐Yi Li, Zuo‐Tao Wu, Yong‐Jie Zhu, Ting Zhuo, Ju‐Sen Nong, Jing Qian, Hua‐Jian Peng, Lei Dai, Yong‐Yong Wang, Ming‐Wu Chen, Xiao‐Chun Zeng

**Affiliations:** ^1^ Department of Cardio‐Thoracic Surgery The First Affiliated Hospital of Guangxi Medical University Nanning Guangxi China; ^2^ Geriatrics Department of Endocrinology and Metabolism The First Affiliated Hospital of Guangxi Medical University Nanning Guangxi China; ^3^ Department of Respiratory Medicine The First Affiliated Hospital of Guangxi Medical University Nanning Guangxi China; ^4^ Department of Pediatric Surgery The First Affiliated Hospital of Guangxi Medical University Nanning Guangxi China

**Keywords:** immune checkpoint, immune infiltration, lung adenocarcinoma, POC1A, prognostic biomarker, tumour mutation burden

## Abstract

Lung adenocarcinoma (LUAD) is characterized by a high incidence rate and mortality. Recently, POC1 centriolar protein A (POC1A) has emerged as a potential biomarker for various cancers, contributing to cancer onset and development. However, the association between POC1A and LUAD remains unexplored. We extracted The Cancer Genome Atlas (TCGA) and the Gene Expression Omnibus (GEO) data sets to analyse the differential expression of POC1A and its relationship with clinical stage. Additionally, we performed diagnostic receiver operator characteristic (ROC) curve analysis and Kaplan–Meier (KM) survival analysis to assess the diagnostic and prognostic value of POC1A in LUAD. Furthermore, we investigated the correlation between POC1A expression and immune infiltration, tumour mutation burden (TMB), immune checkpoint expression and drug sensitivity. Finally, we verified POC1A expression using real‐time quantitative polymerase chain reaction (RT‐qPCR) and immunohistochemistry (IHC). Cell experiments were conducted to validate the effect of POC1A expression on the proliferation, migration and invasion of lung cancer cells. POC1A exhibited overexpression in most tumour tissues, and its overexpression in LUAD was significantly correlated with late‐stage presentation and poor prognosis. The high POC1A expression group showed lower levels of immune infiltration but higher levels of immune checkpoint expression and TMB. Moreover, the high POC1A expression group demonstrated sensitivity to multiple drugs. In vitro experiments confirmed that POC1A knockdown led to decreased proliferation, migration, and invasion of lung cancer cells. Our findings suggest that POC1A may contribute to tumour development by modulating the cell cycle and immune cell infiltration. It also represents a potential therapeutic target and marker for the diagnosis and prognosis of LUAD.

## 1 INTRODUCTION

In the most recent cancer statistics released by the American Cancer Society, the study revealed approximately 2.2 million new cases of lung cancer, which represents the second highest number of new cancer cases, and approximately 1.8 million deaths from lung cancer, signifying the highest death rate among all cancers.^1^ Lung cancer can be categorized into two primary types: non‐small‐cell lung cancer (NSCLC) and small‐cell lung cancer, with NSCLC constituting about 85% of all cases.^2^ Lung adenocarcinoma (LUAD), a subtype of NSCLC, stands as the most prevalent pathological type.^3^ Despite advancements in immunization and targeted therapy, the prognosis for patients with LUAD remains suboptimal, with a reported 5‐year overall survival rate of only 16% according to statistics.^4^ While targeting EGFR, ALK and TKI may enhance the prognosis for LUAD patients, drug resistance poses a significant obstacle, resulting in poor therapeutic outcomes.^5^, ^6^ Hence, it is crucial to explore new effective markers for the early diagnosis and treatment of lung cancer.

POC1 centriolar protein A (POC1A), also known as WDR51A, plays a role in centriole generation and homeostasis, contributing to the composition of centrosomes.^7^ Studies have demonstrated that POC1A is associated with abnormal manifestations of cell mitosis and serves as a regulatory factor in the cell cycle.^8^, ^9^, ^10^ Anomalous cell mitosis can lead to errors in chromosome separation, closely linked to tumorigenesis, cancer prognosis and treatment response.^11^ Research by Hassan Dastsooz et al. suggests that POC1A may influence tumour progression and represent a potential therapeutic target for cancer.^12^ Investigations by Ceyda Kasavi indicate that POC1A exhibits superior diagnostic capability for ovarian cancer and could serve as a candidate factor for diagnosis and treatment.^13^ Yuma Wada et al.'s research identified POC1A as a critical marker for predicting intra‐hepatic cholangiocarcinoma recurrence.^14^ However, the correlation between POC1A and LUAD has not been extensively explored.

Early diagnosis and precise treatment of LUAD are pivotal factors in enhancing patients' quality of life and extending survival. This study thoroughly examined POC1A expression and its implications for diagnosis, staging, prognosis, immune infiltration, tumour mutation burden (TMB), immune checkpoint profiles and drug sensitivity in order to elucidate the clinical significance of POC1A for LUAD.

## 2 MATERIALS AND METHODS

### 2.1 Data acquisition

Long non‐coding RNA (lncRNA) data, messenger RNA (mRNA) expression data (including count and fpkm) and clinical information (comprising stage, survival status and overall survival time) for LUAD as well as the Pan‐Cancer dataset in The Cancer Genome Atlas (TCGA) were obtained from UCSC Xena (xenabrowser.net). Multiple data sets (GSE27262, GSE30219, GSE31210 and GSE43458) for LUAD were downloaded from the Gene Expression Omnibus (GEO) (https://www.ncbi.nlm.nih.gov/geo/)to cross‐validate the results. The acquired data was log‐transformed (log2(x + 1)). Simple nucleotide variation data for LUAD in TCGA was obtained through the Genomic Data Commons (https://portal.gdc.cancer.gov/).

### 2.2 Differential expression of POC1A in different tissues

The R package UCSCXenaShiny was utilized to analyse and visualize the differential expression between various tumours and normal tissues sourced from TCGA. Data from TCGA were utilized to assess the differential expression of POC1A between LUAD and normal samples. The R package ROCR was employed to construct diagnostic receiver operator characteristic (ROC) curves to distinguish tumour and normal samples, thereby evaluating the diagnostic value of POC1A for LUAD. Multiple GEO data sets (GSE27262, GSE30219, GSE31210 and GSE43458) were used to validate the differential expression and diagnostic performance of POC1A. Immunohistochemical images of POC1A in LUAD and lung tissues were obtained from the Human Protein Atlas (HPA) (www.proteinatlas.org) to demonstrate variations in protein expression levels.

### 2.3 Clinical value and survival analysis of POC1A

Differential expression of POC1A among LUAD samples at different stages was compared using data extracted from the TCGA and GSE30219 data sets. Survival analysis was conducted using the R packages survival and survminer to assess the prognostic value of POC1A. LUAD samples were categorized into high‐ or low‐expression groups based on the median expression level of POC1A. KM survival analysis was performed for the total survival time and survival state of TCGA, GSE30219 and GSE31210 data sets, and survival curves were generated.

### 2.4 Functional enrichment analysis of POC1A

Tumour cases with high and low POC1A expression in TCGA and GSE31210 data sets were analysed using the R package limma to identify differentially expressed genes (DEGs) of POC1A. DEGs were filtered using a threshold of the absolute value of log2foldchange ≥1.0. Volcano plots for DEGs obtained from TCGA and GSE31210 data sets were created using R packages ggpubr and ggthemes, respectively. Additionally, Gene Ontology (GO), Kyoto Encyclopedia of Genes and Genomes (KEGG) and Gene Set Enrichment Analysis (GSEA) were conducted for DEGs of the two datasets to explore the functions of POC1A.

### 2.5 Relationship between immune infiltration and POC1A

The Tumour Immune Estimation Resource (TIMER) (https://cistrome.shinyapps.io/timer/) is an online analysis tool^15^ that was utilized to evaluate the relationship between POC1A copy number variation and immune infiltration levels. The expression levels of 28 immune cells in tumour cases were calculated using single‐sample GSEA (ssGSEA) through the R package GSVA. Furthermore, the microenvironment composition of each tumour case was assessed using Estimation of STromal and Immune cells in MAlignant Tumours using Expression data (ESTIMATE) through the R package estimate, which was quantified into Stromal score, immune score, ESTIMATE score and tumour purity. Differences between the two groups with different POC1A expressions (cutoff value set at 50%) were, respectively, compared.

### 2.6 TMB and immune checkpoint expression

The R package maftools were employed to draw and visualize waterfall plots of gene mutations in different POC1A expression groups. TMB and immune checkpoint expression differences between the two groups of tumour samples with different POC1A expressions were compared. The cutoff value for grouping tumour samples based on POC1A expression was set at the quartile.

### 2.7 Connection between drug sensitivity and POC1A

The R package oncoPredict was used to predict the susceptibility of each tumour sample to multiple drugs. Variations in sensitivity to these drugs were compared between the two groups with differing POC1A expression (with the cutoff value set at 50%).

### 2.8 Identification and analysis of hub genes

The R package weighted gene coexpression network analysis (WGCNA) was utilized to conduct WGCNA for the DEGs, resulting in their division into distinct modules. Subsequently, relevant modules associated with clinical phenotypes were screened. Within these specific modules, gene significance (GS) and module membership (MM) were both set at a threshold of 0.8. A protein–protein interaction (PPI) network was then constructed for the identified genes. Ultimately, six closely interacting genes linked with POC1A were identified as hub genes. The expression levels of these hub genes between LUAD and normal samples were analysed, followed by Kaplan–Meier (KM) survival analysis.

### 2.9 Construction of competing endogenous RNA (ceRNA) network

The online tool ENCORI (https://starbase.sysu.edu.cn/) was employed to predict related microRNAs (miRNAs) and lncRNAs. Following the ceRNA theory, predicted miRNAs displaying a negative correlation with POC1A expression (Spearman coefficient *r* < −0.3, *p* < 0.05) were considered as miRNAs targeting POC1A. Core miRNAs of POC1A are defined as those differentially expressed in tumour and normal cases, with their expression significantly impacting the prognosis of tumour cases. In line with the ceRNA theory, predicted lncRNAs inversely correlated with miRNA (Spearman coefficient *r* < −0.3, *p* < 0.05) were considered lncRNAs targeting miRNA. Core lncRNAs also fulfilled the conditions of differential expression between tumour and normal cases, influencing the prognosis of tumour cases.

### 2.10 Real‐time‐quantitative polymerase chain reaction (RT‐qPCR)

The human tissue verification experiment in this study was approved by the Medical Ethics Committee of the First Affiliated Hospital of Guangxi Medical University, and all participants provided written informed consent. Tumours and adjacent tissues from 25 LUAD patients who underwent surgery were obtained from the First Affiliated Hospital of Guangxi Medical University. Total RNA was extracted from these tissues and subsequently reverse transcribed into cDNA. Finally, RT‐qPCR was conducted to analyse POC1A expression using the following primer sequences: POC1A‐F, 5′‐CATTCTGGAGCAGCGGTTGA‐3′; POC1A‐R, 5′‐CCCACAGAGTTCAGTCCCCT‐3′.

### 2.11 Immunohistochemistry (IHC)

Paraffin‐embedded tissue samples of cancer and para‐carcinoma tissues were obtained from three LUAD patients at the First Affiliated Hospital of Guangxi Medical University. Immunohistochemical staining was performed on each tissue according to the instructions provided in the general two‐step kit. The tissue sections were incubated with anti‐POC1A (1:100) overnight at 4°C. Subsequently, they were incubated for 30 min with the enhanced enzyme‐labelled goat anti‐mouse/rabbit IgG polymer. DAB chromogenic kit (ZLI‐9018, Origin, Beijing) and haematoxylin were used for colour development and counterstaining of tissue sections.

### 2.12 Cell culture and RNA interference

Human NSCLC cell lines (A549 and H1299) were acquired from the Chinese Academy of Sciences (Shanghai, China). Dulbecco's Modified Eagle Medium (Gibco, Grand Island, USA) was used to culture A549 and H1299 cells, with the addition of 10% fetal bovine serum (Gibco), penicillin and streptomycin to the medium. These cell lines were maintained at 37°C and 5% CO_2_ and subsequently subjected to small interfering RNA (siRNA) (Nanning Genesis Biotechnology Ltd, China) using Lipofectamine 8000 (Beyotime, China) in a serum‐free medium after 24 h of culture. The siRNA targeting POC1A (si‐POC1A) sequence was 5′‐CAGUGAUGACAAGACUGUUAA‐3′, whereas a non‐targeted siRNA was used with the sequence 5′‐UUCUCCGAACGUGUCACGU‐3′.

### 2.13 Cell proliferation

Cell proliferation assays were performed using the BeyoClickTM EdU‐555 Cell Proliferation Detection Kit (EdU; Beyotime) and Cell Counting Kit‐8 (CCK‐8; Beyotime). After transfection for 24 h, 5‐ethynyl‐2′‐deoxyuridine (EdU) solution was added to the cells, and fluorescence microscopy (EVOS M7000, Thermo Fisher Scientific, USA) was used to observe and photograph the cells. CCK‐8 solution was added at 24, 48 and 72 h, followed by absorbance measurement at 450 nm.

### 2.14 Wound healing assay

Cells were seeded into a six‐well plate, and upon reaching a density of approximately 80%–90%, a vertical wound was created using a 10 μL sterile needle tip. The same area was captured by microscope (Nikon Japan) at 0 and 24 h.

### 2.15 Transwell assay

Transwell chambers (LABSELECT, China) with an 8 μm pore size were utilized for migration and invasion assays. Cells harvested 24 h after transfection were resuspended and added to the upper chamber at a volume of 250 μL. Additionally, 700 μL of medium containing 10% fetal bovine serum (FBS) was placed in the lower chamber, and the system was then incubated for 36 h.

### 2.16 Statistical analysis

Statistical analyses were conducted using R, version 4.2.3. The t‐test was applied to analyse differences between the two groups. KM survival analysis was performed using the log‐rank test. Spearman's correlation was employed for correlation analysis. The threshold for statistical significance was set at *p* < 0.05 (ns, *p* > 0.05, *p < 0.05, **p < 0.01, ***p < 0.001).

## 3 RESULTS

### 3.1 Differential expression of POC1A and its diagnostic value in LUAD

**FIGURE 1:**
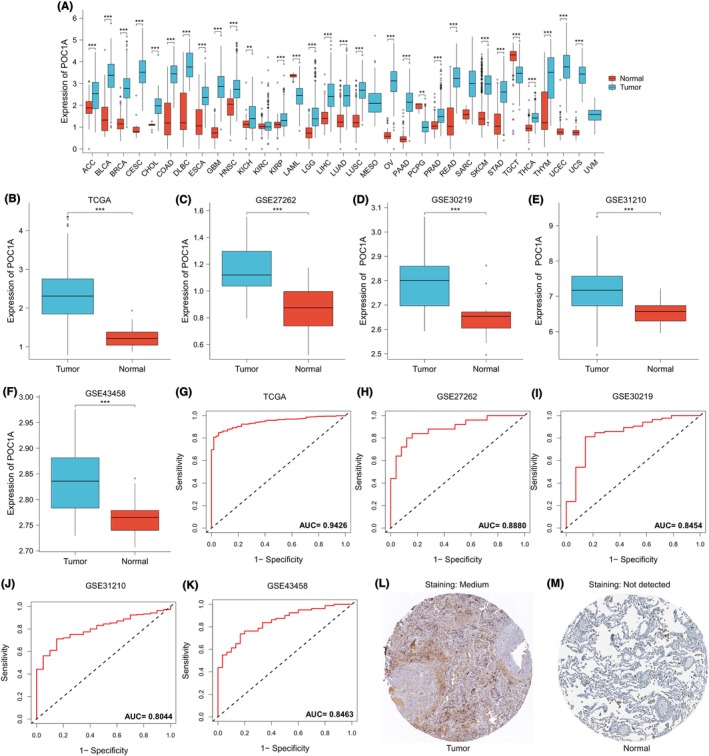
Up‐regulation of POC1A in LUAD. (A) Comparative analysis of POC1A expression differences between tumour samples and normal samples across various cancer types. (B–F) Assessment of differential POC1A expression between tumour and normal groups in TCGA, GSE27262, GSE30219, GSE31210 and GSE43458 data sets. (G–K) Construction of diagnostic ROC curves based on POC1A expression in TCGA, GSE27262, GSE30219, GSE31210 and GSE43458 datasets. (L,M) Immunohistochemical images of POC1A were obtained from the HPA database, including staining in LUAD and normal lung tissue.

The pan‐carcinoma analysis revealed up‐regulation of POC1A across most cancers (Figure 1A). Joint analysis of TCGA and GEO data sets consistently demonstrated increased expression of POC1A in LUAD (Figure 1B–F). The diagnostic ROC curves from TCGA, GSE27262, GSE30219, GSE31210 and GSE43458 exhibited the area under the curve (AUC) values of 0.9426, 0.8880, 0.8454, 0.8044 and 0.8463, respectively, all exceeding 0.8 (Figure 1G–K), indicating the robust diagnostic performance of POC1A for LUAD. Immunohistochemical staining displayed deeper POC1A expression in LUAD compared to normal lung tissue (Figure 1L,M).

### 3.2 Clinical and prognostic value of POC1A

**FIGURE 2:**
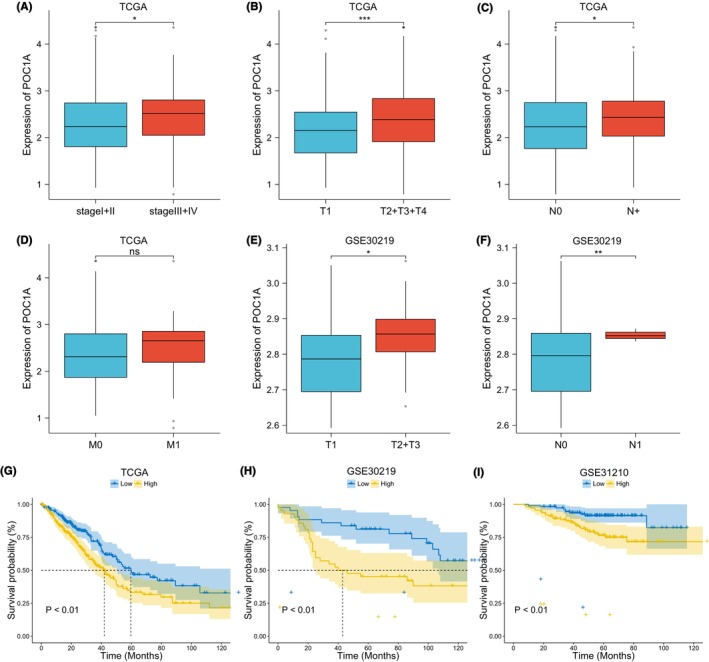
Overexpression of POC1A linked to late‐stage and unfavourable prognosis in LUAD. (A) Comparison of POC1A expression among LUAD samples of different pathological stages in the TCGA data set. (B) Comparison of POC1A expression among LUAD samples of different T stages in the TCGA data set. (C) Analysis of POC1A expression differences between LUAD samples without and with lymph node metastasis (N+) in the TCGA data set. N+: N1, N2, N3 stage. (D) Analysis of POC1A expression among LUAD cases of different M stages in the TCGA data set. (E) Evaluation of POC1A expression at different T stages in GSE30219 data set. (F) Examination of POC1A expression at different N stages in GSE30219 data set. (G–I) KM survival analysis of POC1A in TCGA, GSE30219 and GSE31210 data sets.

Expression analysis revealed up‐regulation of POC1A in samples with higher pathological stage (Figure 2A), T stage (Figure 2B,E) and lymph node metastasis (Figure 2C,F); however, no significant difference was found among different M stages (Figure 2D). KM survival analysis from TCGA (Figure 2G), GSE30219 (Figure 2H), and GSE31210 (Figure 2I) data sets indicated an adverse prognosis associated with POC1A overexpression in LUAD patients.

### 3.3 Functional enrichment analysis of DEGs related to POC1A

**FIGURE 3:**
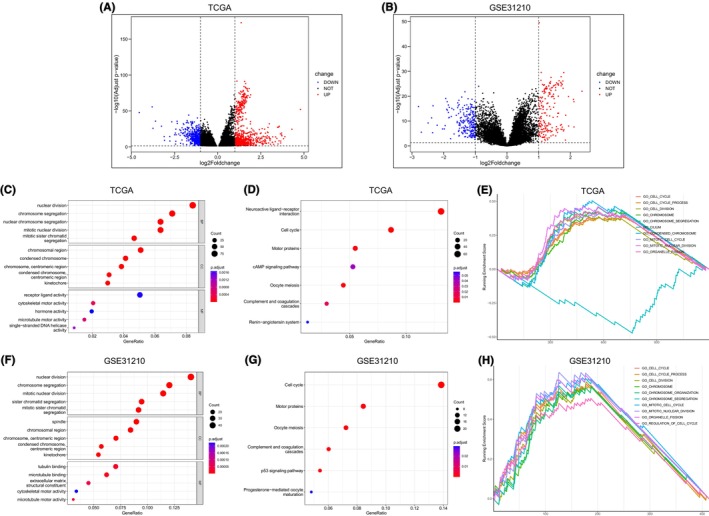
DEGs and functional analysis of POC1A. (A) Volcano map illustrating DEGs obtained from analysis and filtering in TCGA dataset. (B) Volcano map illustrating DEGs obtained from analysis and filtering in GSE31210 data set. (C, D) Dot plots presenting the GO and KEGG analyses based on the TCGA data set. (E) Demonstration of the results obtained by GSEA analysis of DEGs from the TCGA data set. (F–H) Outcomes of GO, KEGG and GSEA analyses of DEGs from the GSE31210 data set.

In the TCGA data set, 1271 DEGs associated with POC1A were identified (Figure 3A), while the GSE31210 data set yielded 414 DEGs (Figure 3B). Subsequently, functional enrichment analyses were independently performed for both groups of DEGs. Biological Process (BP) in GO analysis showed five items, DEGs of TCGA dataset (Figure 3C) and GSE31210 data set (Figure 3F) clustered in four common items, including ‘nuclear division’, ‘chromosome segregation’, ‘mitotic nuclear division’ and ‘mitotic sister chromatid segregation’. With KEGG enrichment analysis, DEGs of TCGA data set were concentrated in these terms, including ‘Neuroactive ligand−receptor interaction’, ‘Cell cycle’, ‘Motor proteins’, ‘cAMP signalling pathway’, ‘Oocyte meiosis’, ‘Complement and coagulation cascades’ and ‘Renin−angiotensin system’ (Figure 3D). On the other hand, the GSE31210 data set emphasized terms including ‘Cell cycle’, ‘Motor proteins’, ‘Oocyte meiosis’, ‘Complement and coagulation cascades’, ‘p53 signalling pathway’ and ‘Progesterone‐mediated oocyte maturation’ (Figure 3G). Moreover, GSEA analysis of the TCGA data set showcased concentration in specific terms such as ‘GO_CELL_CYCLE’, ‘GO_CELL_CYCLE_PROCESS’, ‘GO_CELL_DIVISION’, ‘GO_CHROMOSOME’, ‘GO_CHROMOSOME_SEGREGATION’, ‘GO_CILIUM’, ‘GO_CONDENSED_CHROMOSOME’, ‘GO_MITOTIC_CELL_CYCLE’, ‘GO_MITOTIC_NUCLEAR_DIVISION’ and ‘GO_ORGANELLE_FISSION’ (Figure 3E). The DEGs of GSE31210 data set in GSEA analysis were concentrated in these terms, including ‘GO_CELL_CYCLE’, ‘GO_CELL_CYCLE_PROCESS’, ‘GO_CELL_DIVISION’, ‘GO_CHROMOSOME’, ‘GO_CHROMOSOME_ORGANIZATION’, ‘GO_CHROMOSOME_SEGREGATION’, ‘GO_MITOTIC_CELL_CYCLE’, ‘GO_MITOTIC_NUCLEAR_DIVISION’, ‘GO_ORGANELLE_FISSION’ and ‘GO_REGULATION_OF_CELL_CYCLE’ (Figure 3H).

### 3.4 Correlation between POC1A and immune infiltration

**FIGURE 4:**
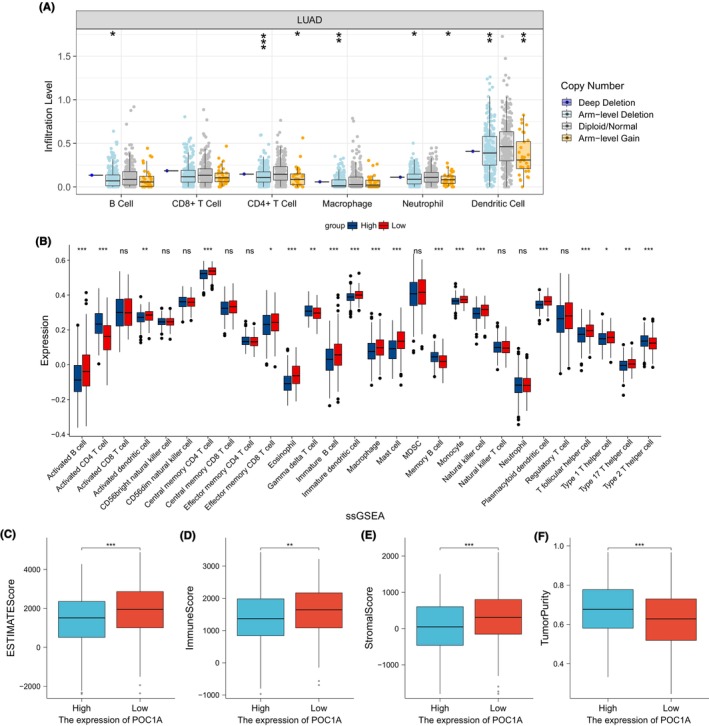
Analysis of immune infiltration in LUAD. (A) Differences in immune infiltration levels between different CNV types of POC1A in LUAD. CNV: copy number variation. (B) Comparison of immune cell expression in LUAD samples with different POC1A expression. (C–F) Assessment of differences in the ESTIMATE, immune, stromal score and tumour purity between two groups with different POC1A expression.

When the arm level of POC1A experienced gain or deletion, a significant reduction in the infiltration levels of B cells, CD4+ T cells, macrophages, neutrophils and dendritic cells was observed (Figure 4A). ssGSEA results revealed a down‐regulation in the expression of 15 subtypes of immune cells in samples exhibiting high POC1A expression. An inverse correlation between POC1A and these immune cells, including activated B cell, activated dendritic cell, central memory CD4 T cell, effector memory CD8 T cell, eosinophil, immature B cell, immature dendritic cell, macrophage, mast cell, monocyte, natural killer cell, plasmacytoid dendritic cell, T follicular helper cell, Type 1 T helper cell and Type 17 T helper cell, was observed (Figure 4B). Additionally, the high POC1A expression group displayed lower ESTIMATE, immune, and stromal scores, but higher tumour purity (Figure 4C–F).

### 3.5 Association of POC1A with gene mutation and immune checkpoint

**FIGURE 5:**
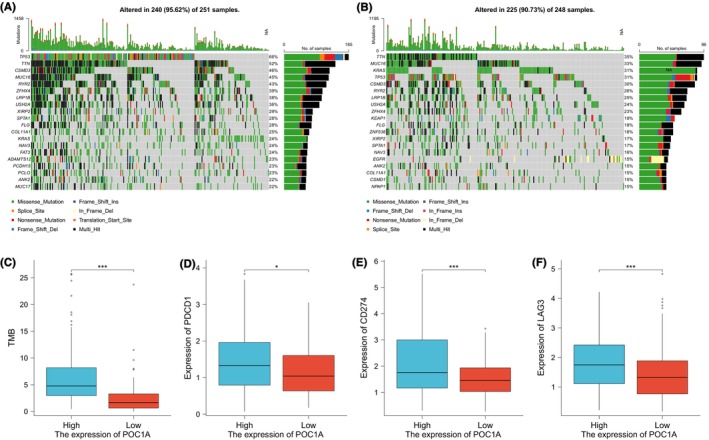
Positive relationship of POC1A with TMB and immune checkpoint. (A) Waterfall diagram depicting gene mutation in high POC1A expression group. (B) Waterfall diagram depicting gene mutation in low POC1A expression group. (C) Difference of TMB between the two groups based on POC1A expression. (D) Comparison of PDCD1 expression between POC1A high and low groups. (E) Comparison of CD274 expression between groups with inconsistent POC1A expression. (F) Comparison of LAG3 expression between groups, based on the median POC1A expression in tumour samples.

The impact of immune checkpoint expression and TMB on the efficacy of immunotherapy within tumour samples underscored the potential correlation between POC1A and immunotherapy. This relationship can be elucidated through exploring their respective associated pathways. The waterfall diagrams illustrated the top 20 genes exhibiting the most mutations in POC1A high‐ (Figure 5A) and low (Figure 5B)‐expression groups, revealing that the proportion of TP53 mutations was notably higher, exceeding 50% in POC1A overexpressed samples. Furthermore, TMB, calculated based on the number of gene mutations, demonstrated a significantly higher value in the POC1A high‐expression group (Figure 5C). Upon comparing the two groups with differing POC1A expressions, the high‐expression group exhibited elevated levels of programmed cell death 1 (PDCD1), CD274 and Lymphocyte‐activation gene 3 (LAG3) (Figure 5D–F).

### 3.6 Analysis of drug sensitivity in LUAD

**FIGURE 6:**
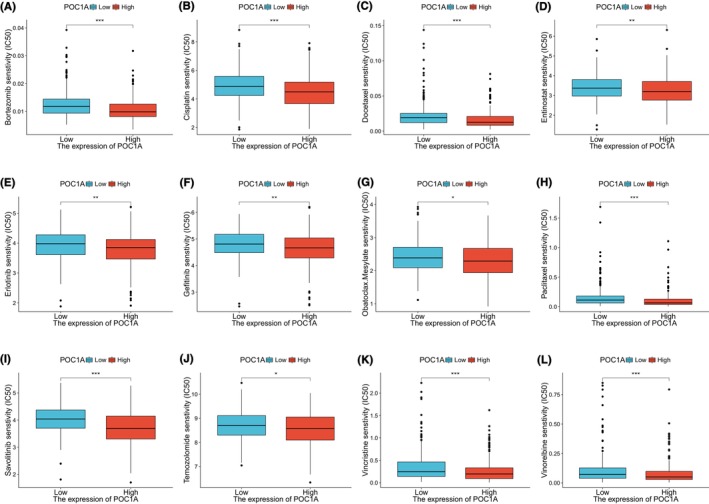
Association between drug sensitivity and POC1A expression. (A) Comparison of bortezomib sensitivity between two groups. (B) Comparison of cisplatin sensitivity. (C) Comparison of docetaxel sensitivity. (D) Comparison of entinostat sensitivity. (E) Comparison of erlotinib sensitivity. (F) Comparison of gefitinib sensitivity. (G) Comparison of obatoclax mesylate sensitivity. (H) Comparison of paclitaxel sensitivity. (I) Comparison of savolitinib sensitivity. (J) Comparison of temozolomide sensitivity. (K) Comparison of vincristine sensitivity. (L) Comparison of vinorelbine sensitivity. LUAD samples were divided into two groups based on POC1A median expression, and drug sensitivity was compared between the two groups.

The group exhibiting high POC1A expression demonstrated lower IC50 values for bortezomib (Figure 6A), cisplatin (Figure 6B), docetaxel (Figure 6C), entinostat (Figure 6D), erlotinib (Figure 6E), gefitinib (Figure 6F), obatoclax mesylate (Figure 6G), paclitaxel (Figure 6H), savolitinib (Figure 6I), temozolomide (Figure 6J), vincristine (Figure 6K) and vinorelbine (Figure 6L). This suggests that LUAD samples overexpressing POC1A may exhibit greater sensitivity to these drugs.

### 3.7 Identification and analysis of hub genes

**FIGURE 7:**
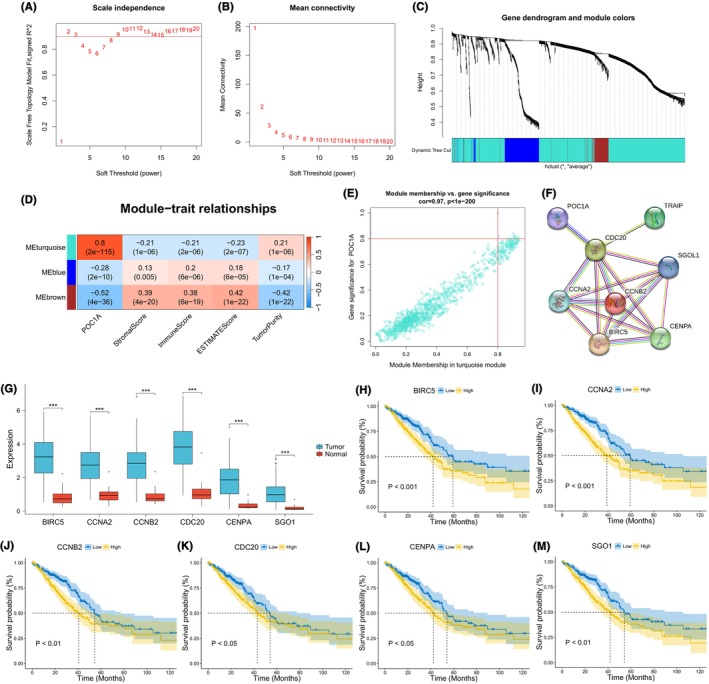
Exploration and analysis of downstream genes of POC1A in LUAD. (A, B) Calculation of scale‐free fitting index and mean connectivity under different soft thresholds (powers). (C) Gene dendrogram of LUAD cases for clustering. (D) Heat map illustrating the relevance of gene modules and clinical traits. (E) Scatter plot depicting gene distribution according to MM and GS values in the turquoise module. (F) PPI networks of genes with MM and GS greater than 0.8 in turquoise modules. (G) Differences in the expression of hub genes between LUAD and normal groups. (H–M) KM survival analysis of hub genes.

In the analysis of 1271 DEGs in TCGA, WGCNA classified these genes into three modules (Figure 7A–C). A strong correlation was observed between the turquoise module and POC1A, thus emphasizing the significance of turquoise module (Figure 7D). Eight genes (BIRC5, CCNA2, CCNB2, CDC20, CENPA, POC1A, SGO1 and TRAIP) from the turquoise module met the criteria with both MM and GS exceeding 0.8 (Figure 7E). Subsequently, a PPI network constructed using the eight genes highlighted six closely interacting genes as hub genes (BIRC5, CCNA2, CCNB2, CDC20, CENPA and SGO1) (Figure 7F). Expression analysis revealed up‐regulation of all hub genes in LUAD samples (Figure 7G), with their overexpression correlating to an unfavourable prognosis for patients (Figure 7H–M).

### 3.8 Establishment of ceRNA networks targeting POC1A

**FIGURE 8:**
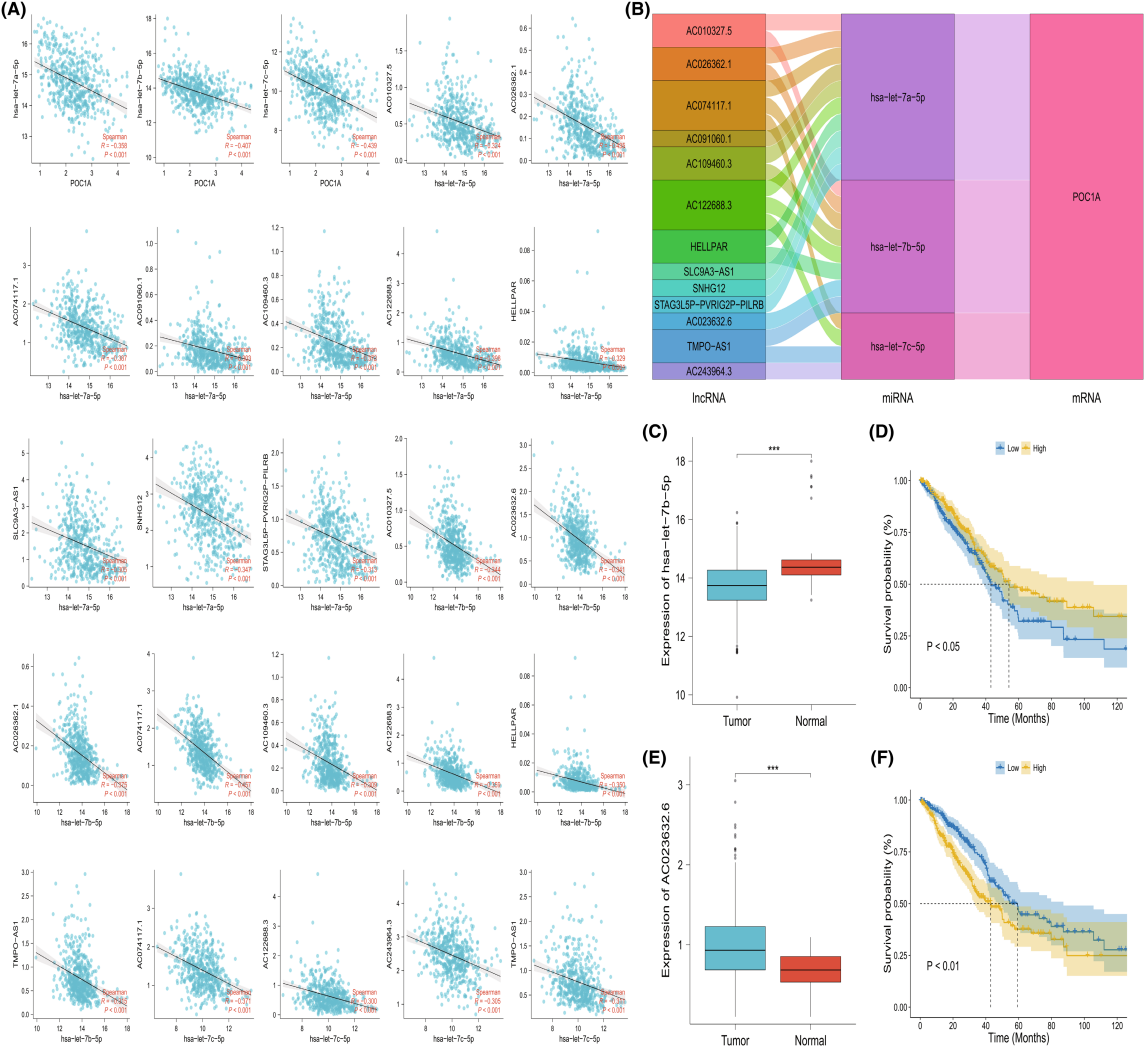
Exploration of upstream genes of POC1A. (A) Correlation analysis between POC1A and miRNAs and between miRNAs and lncRNAs. (B) Sankey diagram representing the ceRNA network for POC1A. (C) Expression difference of core miRNA (hsa−let−7b−5p) in LUAD group and normal group. (D) KM survival analysis of core miRNA (hsa−let−7b−5p) in LUAD samples. (E) Expression difference of core lncRNA (AC023632.6) in LUAD group and normal group. (F) KM survival analysis of core lncRNA (AC023632.6) in LUAD samples.

An inverse correlation was observed between POC1A and three predicted miRNAs (hsa−let−7a−5p, hsa−let−7b−5p, hsa−let−7c−5p). Specifically, hsa−let−7a−5p exhibited an inverse correlation with 10 predicted lncRNAs (AC010327.5, AC026362.1, AC074117.1, AC091060.1, AC109460.3, AC122688.3, HELLPAR, SLC9A3‐AS1, SNHG12 and STAG3L5P‐PVRIG2P‐PILRB), while hsa−let−7b−5p was inversely correlated with eight predicted lncRNAs (AC010327.5, AC023632.6, AC026362.1, AC074117.1, AC109460.3, AC122688.3, HELLPAR and TMPO‐AS1). Furthermore, hsa−let−7c−5p displayed an inverse correlation with four predicted lncRNAs (AC074117.1, AC122688.3, AC243964.3 and TMPO‐AS1) (Figure 8A). A comprehensive ceRNA network was developed to visually illustrate the relationships among mRNA, miRNA and lncRNA (Figure 8B). The expression of hsa−let−7b−5p was found to be down‐regulated in LUAD (Figure 8C), with its overexpression in tumour samples predicting favourable outcomes (Figure 8D). AC023632.6 was observed to be overexpressed in LUAD samples (Figure 8E), and its overexpression in LUAD samples was associated with an unfavourable prognosis (Figure 8F). Based on their significant differential expression and survival analysis, AC023632.6 and hsa−let−7b−5p were identified as core lncRNA and core miRNA, respectively.

### 3.9 Verification of differences in POC1A expression using RT‐qPCR and IHC

**FIGURE 9:**
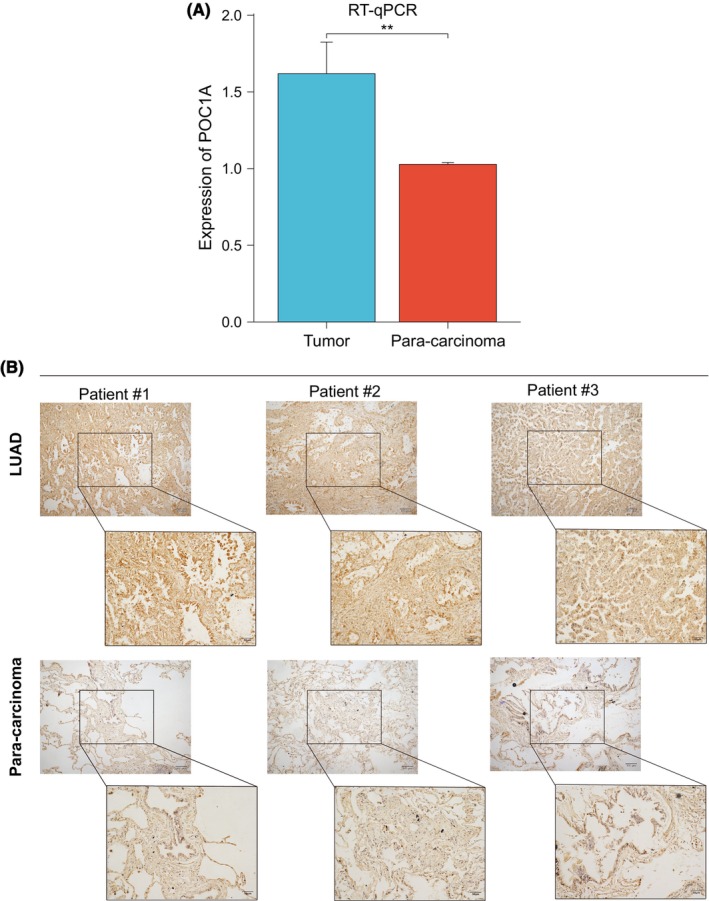
Overexpression of POC1A in LUAD. (A) Validation of POC1A expression in tumour and para‐carcinoma of LUAD patients from Guangxi region using RT‐qPCR. (B) Immunohistochemical staining of tumour tissue and para‐carcinoma lung tissue of three LUAD patients.

RT‐qPCR analysis confirmed higher POC1A expression in tumour tissues compared to paired para‐carcinoma tissues (Figure 9A). Similarly, immunohistochemical staining illustrated a more pronounced POC1A expression in tumour tissue compared to para‐carcinoma lung tissue (Figure 9B).

### 3.10 Functional verification of POC1A in lung cancer cells

**FIGURE 10:**
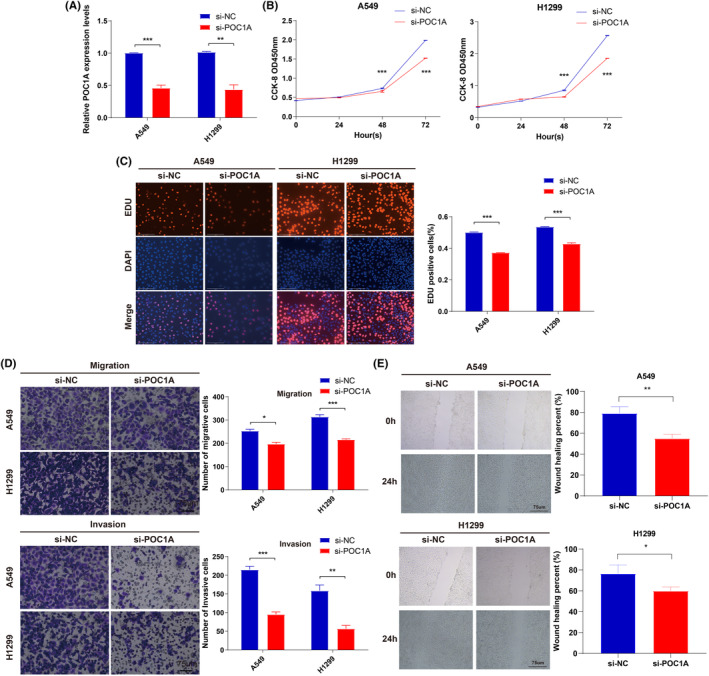
Promotion of proliferation, migration and invasion of lung cancer cells by POC1A. (A) RT‐qPCR of POC1A in A549 and H1299 cells transfected with si‐NC or si‐POC1A. (B) CCK8 assay for the detection of A549 and H1299 proliferation. (C) EdU assay for the detection of A549 and H1299 proliferation. (D) Assessment of invasiveness and migration capability of A549 and H1299 cells via transwell assay. (E) Wound healing assay in si‐NC group and si‐POC1A group from A549 and H1299 cells.

In cell experiments, si‐POC1A was applied to effectively knock down POC1A expression in A549 and H1299 cell lines, aiming to assess the impact of POC1A on lung cancer cell function. RT‐qPCR results confirmed the effective knockdown of POC1A expression in lung cancer cell lines by si‐POC1A (Figure 10A). To investigate the effects of POC1A on lung cancer cell growth, cell counting kit‐8 (CCK8) and EdU assays were conducted, revealing that the knockdown of POC1A expression led to a decrease in cell proliferation (Figure 10B,C). Subsequently, Transwell and wound healing assays were utilized to evaluate the influence of POC1A on lung cancer cell migration, demonstrating that the knockdown of POC1A expression inhibited the migration of lung cancer cells (Figure 10D,E). Additionally, as depicted in Figure 10D, the knockdown of POC1A expression also resulted in the inhibition of invasion by lung cancer cells.

## 4 DISCUSSION

POC1A constitutes a fundamental component of the centrosome, an organelle crucial in cell division and cell cycle regulation.^16^, ^17^ Centrosome amplification serves as a significant hallmark of cancer, and its dysregulated function may signify the initiation and progression of cancer.^18^, ^19^ While existing studies have established an association between POC1A and cancer, its specific correlation with LUAD remains unexplored. As a result, this investigation employed a combined analysis of public datasets and a local cohort to ascertain the significance of POC1A expression in LUAD.

In our investigation, we observed an up‐regulation of POC1A expression across various cancer types, including LUAD. Subsequently, we conducted an analysis to ascertain the disparities in POC1A expression between LUAD samples and normal samples using multiple public datasets. These findings were further validated through RT‐qPCR and immunohistochemical results, confirming significantly higher levels of POC1A mRNA and protein expressions in LUAD compared to normal cases. Furthermore, we utilized POC1A expression as a differentiator for LUAD samples and constructed a diagnostic ROC curve, demonstrating its exceptional diagnostic performance. Examination of different stages of LUAD cases revealed elevated POC1A expression in advanced stages. Recent studies have revealed a close association between heightened POC1A levels and poor prognosis across various cancers, positioning POC1A as an adverse prognostic marker in pan‐cancer.^20^ Consequently, we conducted a survival analysis on POC1A, concluding that high expression of POC1A was indicative of an inferior prognosis compared to the low‐expression group. Based on the aforementioned analysis, it is evident that POC1A may serve as a significant marker for diagnosing and prognosticating LUAD.

The molecular function of POC1A was thoroughly examined using GO, KEGG and GSEA. All functional analyses consistently indicated a close association between POC1A and the cell cycle. Given that the aberrant regulation of the cell cycle is a fundamental characteristic of tumours and is intricately linked to cancer onset and progression,^21^ it is plausible that POC1A may play a role in driving the advancement of LUAD through its influence on the cell cycle. Notably, the KEGG results revealed enrichment of DEGs from TCGA and GSE31210 datasets in the cAMP and p53 signalling pathways, respectively. Furthermore, the cell function experiments conducted in this study unequivocally demonstrated the capacity of POC1A in enhancing the proliferation, migration and invasion of lung cancer cells.

The investigation of the tumour microenvironment holds promise in unravelling the clinical attributes and invasive potential of cancer, with immune infiltration playing a pivotal role in tumour progression.^22^ Our study revealed that variations at the arm level of POC1A influence the levels of infiltrating B cells, CD4+ T cells, macrophages, neutrophils and dendritic cells. Subsequent analysis of the association between POC1A and immune cell infiltration using ssGSEA exposed diminished infiltration of diverse immune cells in samples exhibiting POC1A overexpression. Specifically, 15 subtypes of immune cells exhibited a negative correlation with POC1A, encompassing B cells, dendritic cells, CD4 T cells, CD8 T cells, eosinophils, macrophages, mast cells, monocytes, natural killer cells, T follicular helper cells, Type 1 T helper cells and Type 17 T helper cells. It is noteworthy that B cells foster T cell response and participate in anti‐tumour action by differentiating into plasma cells responsible for producing anti‐tumour antibodies.^23^ Dendritic cells, serving as crucial antigen‐presenting cells, bolster anti‐tumour immunity by activating T cells.^24^ CD8 T cells stand as principal effector cells in anti‐tumour immunity, whereas CD4 T cells play an irreplaceable role in preserving the anti‐tumour properties of CD8 T cells.^25^, ^26^ The function of certain immune cells in either combating or promoting tumours is contingent on various factors; for instance, distinct subtypes of macrophages, M1 and M2, assume opposing roles in tumour immunity.^27^ Additionally, mast cells exhibit anti‐tumour effects in lung cancer but may potentially assume a tumour‐promoting role in other cancer types.^28^, ^29^ Moreover, natural killer T cells can reinvigorate the effector function of deactivated CD8 T cells after stimulation, thus enhancing the anti‐tumour response.^30^ Furthermore, ESTIMATE analysis unveiled that LUAD samples exhibiting high expression of POC1A showcased lower ESTIMATE, immune and stromal scores, although higher tumour purity. Evaluation of the immune microenvironment within LUAD samples indicated that individuals with POC1A overexpression exhibited reduced immune cell infiltration and impaired immune function.

Subsequently, we conducted an analysis of the gene mutation landscape between the two groups exhibiting distinct levels of POC1A expression, revealing that samples with elevated POC1A expression manifested a higher TMB in comparison to those with low expression. TMB serves as a gauge for the quantity of tumour mutations, which are processed as antigens and presented to T cells. A heightened TMB can generate more neoantigens, amplifying the likelihood of T cell recognition and potentially enhancing patients' response to treatment with immune checkpoint inhibitors.^31^ The combination of PDCD1 (PD‐1) and CD274 (PD‐L1) can disrupt the immune environment, culminating in immune evasion by tumour cells, and these molecules can be targeted for therapeutic intervention using immune checkpoint inhibitors.^32^, ^33^ Furthermore, LAG3 has been recognized as a target for co‐inhibitory receptors.^34^ Prior research has demonstrated that combined immunotherapy‐targeting anti‐PD‐1/anti‐LAG3 exerts a superior inhibitory effect on tumour growth in contrast to monotherapy.^35^ Consequently, it is plausible that samples characterized by heightened POC1A expression may exhibit increased responsiveness to immunotherapy.

Subsequently, we examined the association between POC1A and several anti‐tumour medications, encompassing bortezomib, cisplatin, docetaxel, entinostat, erlotinib, gefitinib, obatoclax mesylate, paclitaxel, savolitinib, temozolomide, vincristine and vinorelbine. Our analysis revealed that the half‐maximal inhibitory concentration (IC50) of these drugs was significantly lower in the high POC1A expression group. Bortezomib exhibits remarkable potential in modulating the expression of apoptosis‐related proteins and can be employed in combination with cytotoxic drugs for treating lung cancer.^36^ Cisplatin stands as the most commonly utilized platinum‐based drug, forming the foundation of first‐line chemotherapy for LUAD.^37^, ^38^ In the context of NSCLC treatment, both docetaxel and paclitaxel can be combined with platinum to form a classical chemotherapy regimen, demonstrating favourable efficacy.^39^ Erlotinib is indicated for the treatment and maintenance of second‐ and third‐line advanced NSCLC, while entinostat, in combination with erlotinib, enhances the single‐agent efficacy of erlotinib.^40^ Gefitinib, an epidermal growth factor receptor (EGFR) (EGFR)‐TKI, is widely used in the treatment of LUAD‐carrying EGFR‐activating mutations.^41^ Obatoclax mesylate has exhibited efficacy in treating NSCLC, and its antitumor effect is achieved through the inhibition of Bcl‐2 signalling.^42^ Savolitinib is currently approved for the treatment of metastatic NSCLC with MET exon 14 mutation.^43^ In patients with brain metastases from NSCLC, radiotherapy combined with temozolomide has shown greater effectiveness than radiotherapy alone in safeguarding neurocognitive function.^44^ These findings collectively suggest that individuals exhibiting POC1A overexpression may experience improved outcomes when treated with these drugs.

To investigate the downstream genes of POC1A, we applied WGCNA and constructed a PPI network. Ultimately, we identified six hub genes (BIRC5, CCNA2, CCNB2, CDC20, CENPA and SGO1). Teng Ma et al. independently established that the overexpression of BIRC5 serves as a significant factor associated with a poor prognosis in LUAD, promoting tumour development via the regulation of immune checkpoint expression, leading to immune evasion.^45^ Jun Shan Ruan et al. documented that the up‐regulation of CCNA2 is correlated with an unfavourable prognosis in NSCLC and is implicated in tumour metastasis processes.^46^ Xiyong Wang et al. observed overexpression of CCNB2 in LUAD and proposed its consideration as a prognostic marker for adverse outcomes.^47^ Hao Hu et al. reported the up‐regulation of CDC20 expression in NSCLC and demonstrated its association with poor prognosis.^48^ Haomiao Zhou et al. highlighted the critical role of CENPA in the development of LUAD.^49^ Yixiao Yuan et al. revealed the overexpression of SGO1 in LUAD, which was linked to an unfavourable prognosis.^50^ The differential expression of hub genes and their impact on prognosis underscored the collaborative effect of POC1A and hub genes in advancing the progression of LUAD.

The ceRNA network encompasses both mRNA and non‐coding RNA, collectively involved in competitive regulation. Any aberrant expression from within this network may significantly contribute to the onset and progression of cancer.^51^ According to ceRNA theory, miRNAs can bind to mRNA, thereby inhibiting mRNA expression, and lncRNA can adsorb miRNA to reduce the interference of miRNAs to mRNA, thereby regulating tumour progression.^51^, ^52^ Three upstream miRNAs of POC1A, namely hsa−let−7a−5p, hsa−let−7b−5p and hsa−let−7c−5p, have been predicted, and these miRNAs are utilized to predict their respective upstream lncRNAs. Subsequently, ceRNA networks are established based on their associations. Core lncRNA AC023632.6 and core miRNA hsa−let−7b−5p were identified due to their significant differential expression and subsequent survival analysis.

Nevertheless, it is important to acknowledge the limitations of this study. Firstly, the sample size from the public database may not be sufficiently large, potentially leading to inevitable errors. Secondly, the direct mechanism of POC1A in tumour immunity has not been conclusively confirmed, and its relationship with drug sensitivity remains a theoretical prediction which may necessitate validation through animal experiments and clinical trials.

## 5 CONCLUSIONS

POC1A potentially contributes to tumour development by influencing the cell cycle and immune cell infiltration. As a result, it stands as a promising therapeutic target and a candidate marker for the diagnosis and prognosis of LUAD.

## AUTHOR CONTRIBUTIONS

**Zi‐Hao Li:** Conceptualization (equal); methodology (equal); writing – original draft (equal). **Jia‐Yi Li:** Data curation (equal); software (equal); writing – original draft (equal). **Zuo‐Tao Wu:** Conceptualization (equal); software (equal). **Yong‐Jie Zhu:** Data curation (equal). **Ting Zhuo:** Investigation (equal). **Ju‐Sen Nong:** Software (equal). **Jing Qian:** Formal analysis (equal). **Hua‐Jian Peng:** Formal analysis (equal). **Lei Dai:** Investigation (equal). **Yong‐Yong Wang:** Writing – review and editing (equal). **Ming‐Wu Chen:** Project administration (equal). **Xiao‐Chun Zeng:** Project administration (equal); writing – review and editing (equal).

## FUNDING INFORMATION

This study was funded by grants from the Applied Project of Guangxi Health and Medical Appropriate Technology in Development and Promotion (grant number: S2019105; Recipient: Prof. Xiao‐Chun Zeng).

## CONFLICT OF INTEREST STATEMENT

The authors declare that they have no competing interests.

## CONSENT FOR PUBLICATION

Not applicable.

## Data Availability

The data sets from TCGA and GEO in this study can be found in online databases. R code and experimental data could be obtained from the corresponding author.
